# Transauricular vagus nerve stimulation for patients with disorders of consciousness: A randomized controlled clinical trial

**DOI:** 10.3389/fneur.2023.1133893

**Published:** 2023-03-02

**Authors:** Yi-Fan Zhou, Jun-Wei Kang, Qi Xiong, Zhen Feng, Xiao-Yang Dong

**Affiliations:** Department of Rehabilitation Medicine, The First Affiliated Hospital of Nanchang University, Nanchang, Jiangxi, China

**Keywords:** transauricular vagus nerve stimulation, disorders of consciousness, Coma Recovery Scale-Revised, minimally conscious state (MCS), vegetative state/unresponsive wakefulness syndrome (VS/UWS)

## Abstract

**Introduction:**

Disorders of consciousness (DoCs) are a frequent complication of brain injury disease, and effective treatments are currently lacking. Transauricular vagus nerve stimulation (tVNS) has been proposed as a promising therapeutic method for neurological disorders such as epilepsy and depression. In our previous study, we demonstrated that vagus nerve stimulation promoted recovery in rats with DoCs caused by traumatic brain injury. However, the clinical effect of vagus nerve stimulation on consciousness disorders is unclear. We aimed to investigate the therapeutic efficacy and safety of tVNS in patients with DoCs.

**Methods:**

We conducted a randomized, double-blinded, sham-controlled trial. Patients (N = 60) with DoCs, including minimally conscious state (MCS) and vegetative state/unresponsive wakefulness syndrome, were enrolled and randomized to groups receiving either active or sham tVNS. A frequency of 20 Hz and pulse wave of 200 us was used in the active-tVNS protocol, which was performed in the auricular branch of the vagus nerve in the left outer ear. The sham-tVNS protocol was the same as the active-tVNS protocol although without current input. Both groups of patients also received conventional treatments. Consciousness was evaluated according to the Coma Recovery Scale-Revised before and after the 4-week intervention. We also recorded the type and number of behavioral responses. Safety was primarily assessed according to the incidence of treatment-emergent adverse events. Each patient's heart rate and blood pressure were monitored during all treatment sessions.

**Results:**

Ultimately, 57 patients completed the study: 28 patients underwent active tVNS and 29 patients underwent sham tVNS. No significant differences were observed in Coma Recovery Scale-Revised scores between the active- and sham-tVNS groups before the tVNS sessions. Compared with patients in the sham-tVNS group (9.28 ± 4.38), patients with DoCs treated with active tVNS showed improved consciousness (10.93 ± 4.99), although not statistically significant. Further analysis revealed obvious differences between patients with MCS receiving active and sham tVNS, but no significant difference in patients with vegetative state/unresponsive wakefulness syndrome in both groups. All side effects were considered common medical conditions with no obvious correlation to tVNS.

**Conclusion:**

These preliminary data provide early evidence that tVNS may be an effective and safe approach for promoting the recovery of consciousness, especially in patients with MCS.

**Clinical trial registration:**

https://www.chictr.org.cn/edit.aspx?pid=175938&htm=4, identifier: ChiCTR2200066629.

## 1. Introduction

Disorders of consciousness (DoCs), including minimally conscious state (MCS) and vegetative state/unresponsive wakefulness syndrome (VS/UWS), are characterized by alterations in arousal or awareness. Common causes of DoCs include traumatic brain injury, ischemic stroke, and cardiac arrest (1, 2). With advancements in modern advanced medicine, patients with DoCs have begun to be raised in the worldwide. DoCs are a major global health and socio-economic concern, affecting people of all ages with high mortality and severe disability risks, and also impose a significant economic burden on society and families (3, 4). Family members and caregivers desire effective interventions to speed up the recovery of consciousness with more favorable outcomes for patients with DoCs.

Most treatment regimens performed in patients with DoCs have focused on accelerating clinical recovery through pharmacological interventions, environmental stimulation or, more recently, neuromodulatory brain stimulation techniques (5, 6). Vagus nerve stimulation is a neuromodulation technique that modulates functional brain activity *via* electrical stimulation of the vagus nerve. Recently, experts have focused on the use of vagus nerve stimulation in patients with DoCs. Table 1 clearly shows that previous studies, including two case reports and one case series, have increasingly focused on vagus nerve stimulation for consciousness recovery after brain injures since it was developed in 2017. Experts from Germany, China, and Spain reported that electrical vagus nerve stimulation accelerated the awakening of patients with DoCs (7–9). The vagus nerve is targeted for stimulation based on its effects on cortical plasticity and role in activating the ascending reticular activating system, by which the state of wakefulness is maintained. New mechanisms were also recently identified, such as increased cerebral blood flow and neurotransmitter regulation (10).

**Table 1:** Overview of the studies on vagus nerve stimulation for disorders of consciousness.

**References**	**Type of study**	**Patient**	**Intervention**	**Results**
Yu et al. (7)	Case report	Cardiopulmonary resuscitation, VS	tVNS, twice daily for 30 mins, 4–6 mA, 20 Hz	CRS-R from 6 to 13 after 4 weeks
Corazzol et al. (8)	Case report	Lesion in brains, VS/UWS	VNS, 1.5 mA	CRS-R from 5 to 10
Noe et al. (9)	Case series	6 VS, 8 MCS	tVNS, twice a day, 5 days per week, 4 weeks, 250 us, 20 Hz, 2 mA	CRS-R of five MCS patients improved

VNS, vagus nerve stimulation; tVNS, transcutaneous vagus nerve stimulation; MCS, minimally conscious state; VS, vegetative state; CRS-R, Coma Recovery Scale-Revised.

Although previous studies have demonstrated benefits associated with vagus nerve stimulation, significant limitations were noted, including lack of randomized controlled clinical trials and small sample sizes. Therefore, the primary aim of the present study was to assess the efficacy and safety of transauricular vagus nerve electrical stimulation (tVNS) in promoting consciousness recovery in patients with DoCs using a prospective controlled design.

## 2. Materials and methods

### 2.1. Participants

This randomized, double-blinded, single-center, controlled trial used a parallel design. Patients diagnosed with DoCs were recruited for our study from November 2021–October 2022. We included patients: (1) classified as VS/UWS and MCS based on the Coma Recovery Scale-Revised (CRS-R), which includes six subscales addressing the auditory, visual, motor, verbal, communication, and arousal processes (11); (2) with DoCs for more than 1 month; (3) aged 18–75 years without sex restriction; (4) without central nervous system excitatory drugs use; (5) with their first brain injury; and (6) with no history of other neurological/psychiatric disorders. We excluded patients with: (1) a history of uncontrolled seizures within 1 month; (2) implanted metal object or pacemaker; (3) low pulse rate (pulse rate <60 beats/min); and (4) serious complications, including heart or renal failure. Patients and their legal guardians were given information regarding the procedures of this study; all participants provided written informed consent.

### 2.2. Randomization

We used computerized random number generators for randomization. Half of the numbers were allocated to the active-tVNS group and the other half to the sham-tVNS group. The group assignments were placed in a sealed opaque envelope and randomly distributed to the participants. An independent researcher performed the randomization and did not reveal the assignments until the end of the study. Only the researchers conducting the tVNS intervention were aware of the assignments.

### 2.3. tVNS treatment protocol

Patients in the active-tVNS group received tVNS in addition to conventional treatments. An electrical stimulator (tVNS501, Changzhou Rishena Medical Device Co., Ltd., Jiangsu, China) was applied using the following experimental stimulation parameters: sinusoidal waveform; pulse width, 200 us; frequency, 20 Hz; and intensity, 15 (based on previous studies) (9). tVNS was performed for 30 mins twice daily 6 days per week for 4 weeks. Patients in the sham-tVNS group also underwent a tVNS procedure, although without current output, and received conventional treatments such as multimodal sensory and auditory stimulations, bedside conventional physical therapy, and environmental enrichment therapy in accordance with the current guidelines (12, 13).

### 2.4. Outcome measures

Consciousness was assessed using the CRS-R, a standardized tool to detect subtle changes in neurobehavioral states, before the first and after the last tVNS sessions. CRS-R includes six subscales that assess auditory, visual, motor, and oral motor/speech functions, as well as communication and arousal. Each CRS-R subscale score is based on the presence or absence of a specific behavioral response to sensory stimuli. The total score ranges from 0 to 23, with higher scores indicating better awareness in patients with DoCs. We define a CRS-R increase by one point as “minimally improved,” two points as “improved,” and three or more points as “much improved.” Researchers who assessed consciousness in all patients with DoCs were blinded to the intervention conditions. In addition, each patient's heart rate, breathing, pulse and blood pressure were monitored during all treatment sessions.

### 2.5. Statistical analysis

A paired *t*-test or Wilcoxon test was used to analyze differences in quantitative indicators by comparing CRS-R scores before and after tVNS within groups. The independent sample *t*-test and Mann-Whitney U test were used to compare differences in quantitative indicators between groups. The chi-square test was applied to examine differences in sex and etiology. Statistical significance was set at *P* < 0.05. All data analyses were performed using SPSS software version 25.0 (IBM Corp., Armonk, NY, USA).

## 3. Results

### 3.1. Enrollment and characteristics of the patients

**Figure 1:**
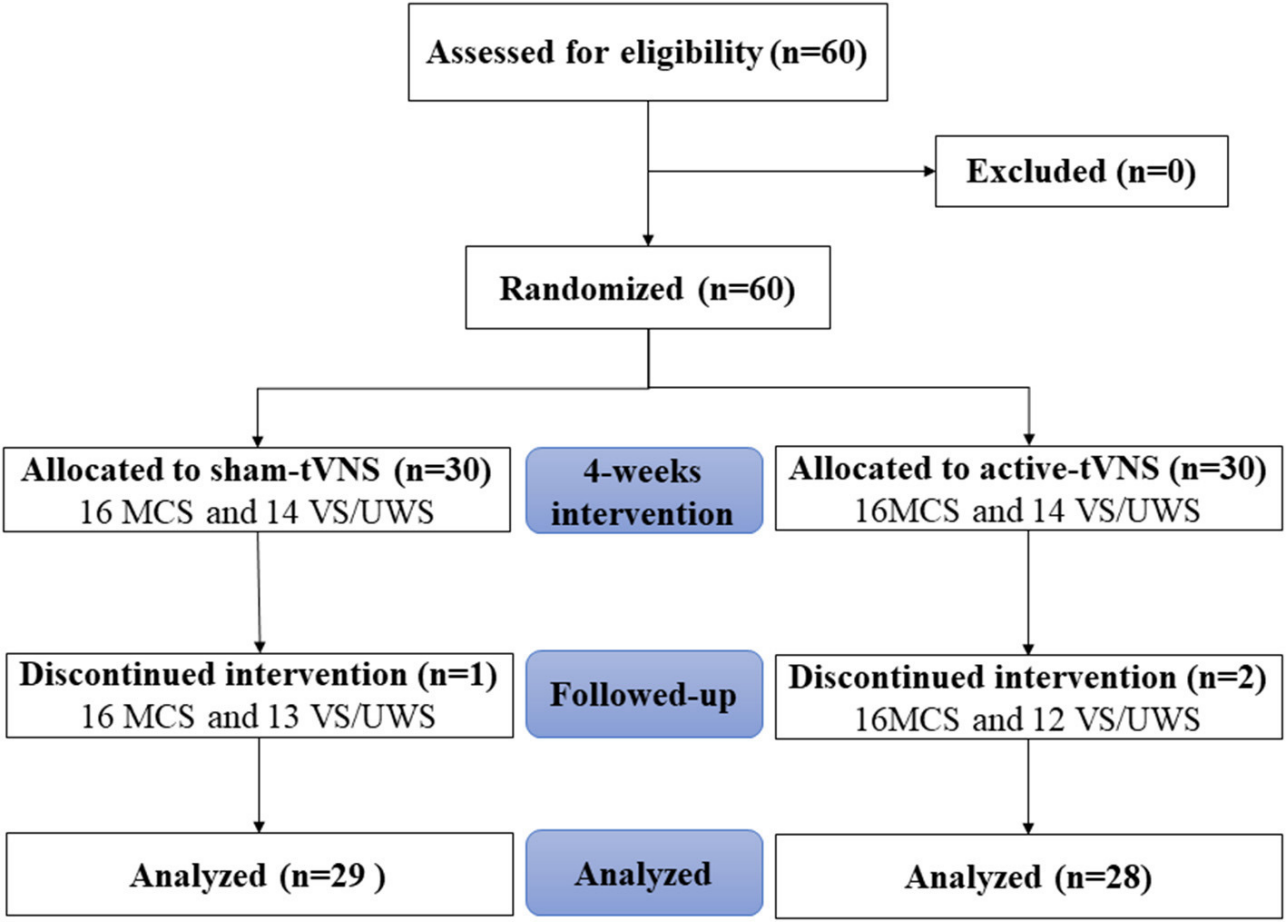
CONSORT flow diagram.

Sixty patients were enrolled in this study; two patients in the active-tVNS group and one in the sham-tVNS group withdrew because of discharge. Eventually, 57 patients with DoCs completed the study (Figure 1). Twenty-eight patients underwent active tVNS and 29 patients underwent sham tVNS. No significant differences in age, sex, etiology, disease duration, and CRS-R scores were observed between the two groups (Table 2). Moreover, no significant differences were observed in the clinical characteristics of patients with MCS and VS/UWS between groups (Tables 3, 4).

**Table 2:** Baseline demographic and clinical comparisons of subjects undergoing active or sham tVNS.

**Variable**	**Active-tVNS group**	**Sham-tVNS group**	***P*-value**
Number	28	29	
Age (year)	55.96 ± 9.83	57.28 ± 9.40	0.609
**Gender**			0.503
Male	16/28 (57.14%)	14/29 (48.28%)	
Female	12/28 (42.86%)	15/29 (51.72%)	
**Etiology**			0.230
Stroke	17/28 (60.71%)	13/29 (44.83%)	
TBI	11/28 (39.29%)	16/29 (55.17%)	
Duration of disease (days)	117.89 ± 15.91	124.31 ± 19.01	0.173
**Clinical assessment**			0.881
VS/UWS	12/28 (42.86%)	13/29 (44.83%)	
MCS	16/28 (57.14%)	16/29 (55.17%)	
CRS-R total score	8.96 ± 4.02	8.72 ± 4.23	0.872
**Surgery**			0.786
Yes	23/28 (82.14%)	23/29 (79.31%)	
No	5/28 (17.86%)	6/29 (20.69%)	

Duration: time at which the patient has been in a disorder of consciousness.

tVNS, transcutaneous vagus nerve stimulation; TBI, traumatic brain injury; MCS, minimally conscious state; VS/UWS, vegetative state/unresponsive wakefulness Syndrome; CRS-R, Coma Recovery Scale-Revised.

**Table 3:** Baseline demographic and clinical comparisons of MCS subjects undergoing active or sham tVNS.

**Variable**	**Active-tVNS group**	**Sham-tVNS group**	***P*-value**
Number	16	16	
Age (year)	55.31 ± 9.46	59.38 ± 9.03	0.223
**Gender**			0.157
Male	10/16 (62.50%)	6/16 (37.50%)	
Female	6/16 (37.50%)	10/16 (62.50%)	
**Etiology**			0.476
Stroke	10/16 (62.50%)	8/16 (50.00%)	
TBI	6/16 (37.50%)	8/16 (50.00%)	
Duration of disease (days)	119.88 ± 11.46	125.19 ± 19.75	0.403
CRS-R total score	12.06 ± 1.98	12.13 ± 2.16	0.933
**Surgery**			1.000
Yes	14/16 (87.50%)	14/16 (87.50%)	
No	2/16 (12.50%)	2/16 (12.50%)	

Duration: time at which the patient has been in a disorder of consciousness.

tVNS, transcutaneous vagus nerve stimulation; TBI, traumatic brain injury; MCS, minimally conscious state; VS/UWS, vegetative state/unresponsive wakefulness Syndrome; CRS-R, Coma Recovery Scale-Revised.

**Table 4:** Baseline demographic and clinical comparisons of VS/UWS subjects undergoing active or sham tVNS.

**Variable**	**Active-tVNS group**	**Sham-tVNS group**	***P*-value**
Number	12	13	
Age (year)	56.83 ± 10.67	54.69 ± 9.56	0.603
**Gender**			
Male	6/12 (50.00%)	8/13 (61.54%)	0.561
Female	6/12 (50.00%)	5/13 (38.46%)	
**Etiology**			0.320
Stroke	7/12 (58.33%)	5/13 (38.46%)	
TBI	5/12 (41.67%)	8/13 (61.54%)	
Duration of disease (days)	115.25 ±16.81	123.23 ± 18.79	0.276
CRS-R total score	4.83 ± 1.34	4.60 ± 1.40	0.577
**Surgery**			0.748
Yes	9/12 (75.00%)	9/13 (69.23%)	
No	3/12 (25.00%)	4/13 (30.77%)	

Duration: time at which the patient has been in a disorder of consciousness.

tVNS, transcutaneous vagus nerve stimulation; TBI, traumatic brain injury; MCS, minimally conscious state; VS/UWS, vegetative state/unresponsive wakefulness Syndrome; CRS-R, Coma Recovery Scale-Revised.

### 3.2. The effects of tVNS on clinical assessment

**Figure 2:**
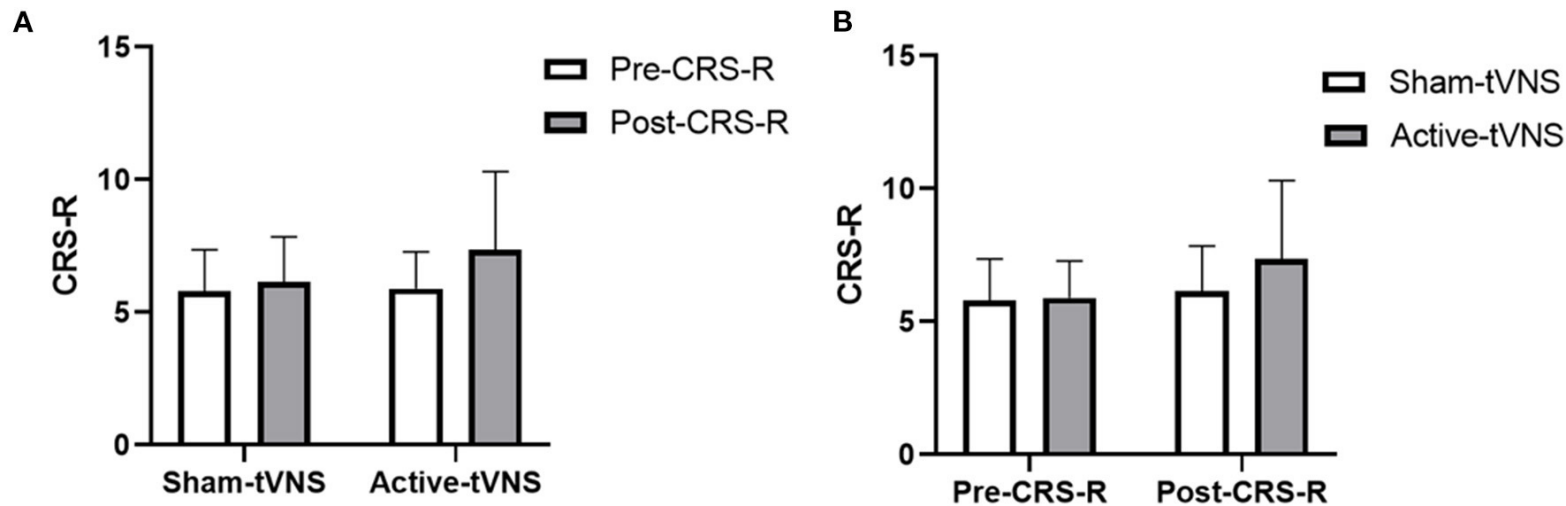
**(A)** Coma Recovery Scale-Revised (CRS-R) improvement before and after treatment in the groups. **(B)** Comparison of CRS-R improvement between the two groups before and after treatment.

**Figure 3:**
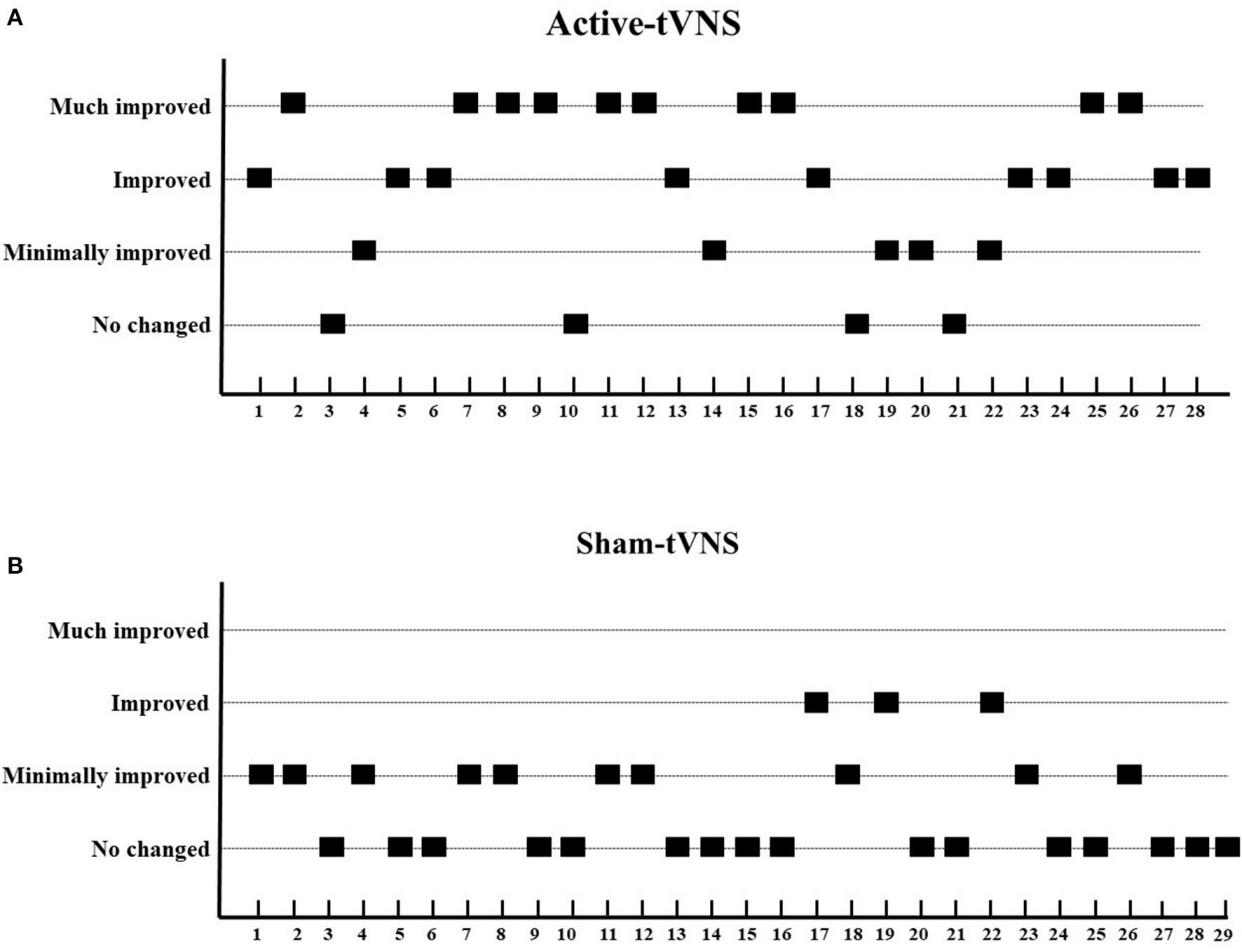
Improvements in consciousness level between two groups. **(A)** Active-tVNS group, **(B)** Sham-tVNS group. We define a CRS-R increase by one point as “minimally improved,” two points as “improved,” and three or more points as “much improved.” Compared with the sham-tVNS group, the active-tVNS group showed greater improvements in consciousness.

**Figure 4:**
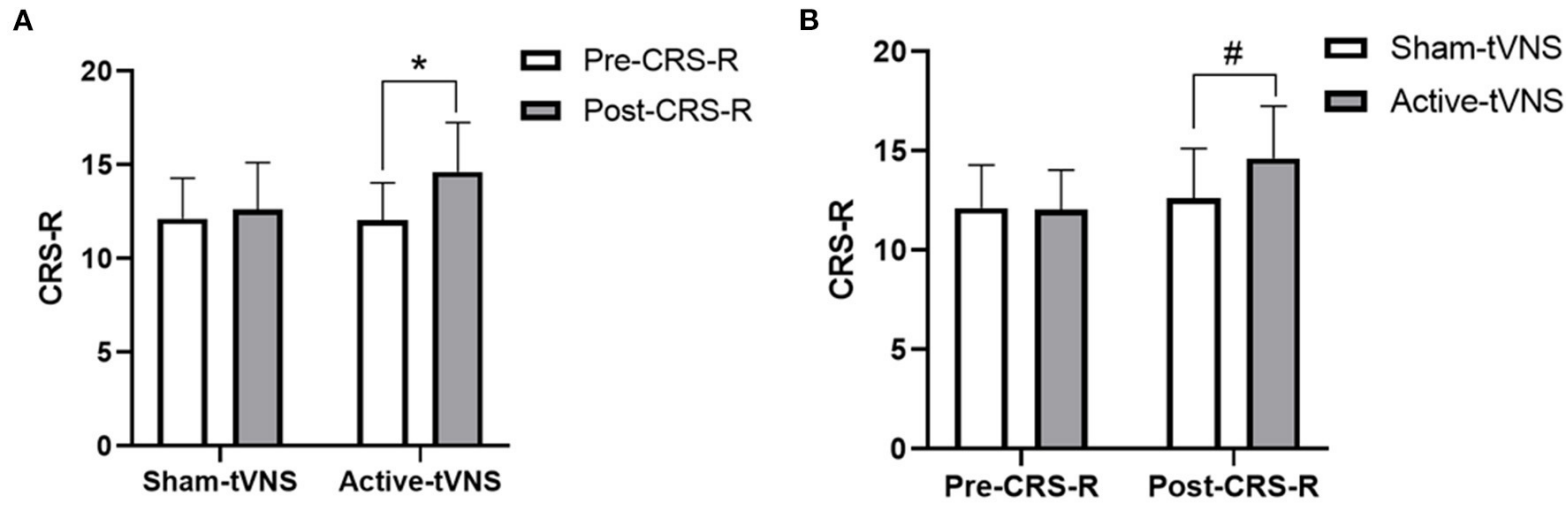
**(A)** Coma Recovery Scale-Revised (CRS-R) improvement in MCS subject before and after treatment in the groups. **(B)** Comparison of CRS-R improvement MCS subject between the two groups before and after treatment. ^*^p < *0.05*, ^#^*p* < *0.05*.

**Figure 5:**
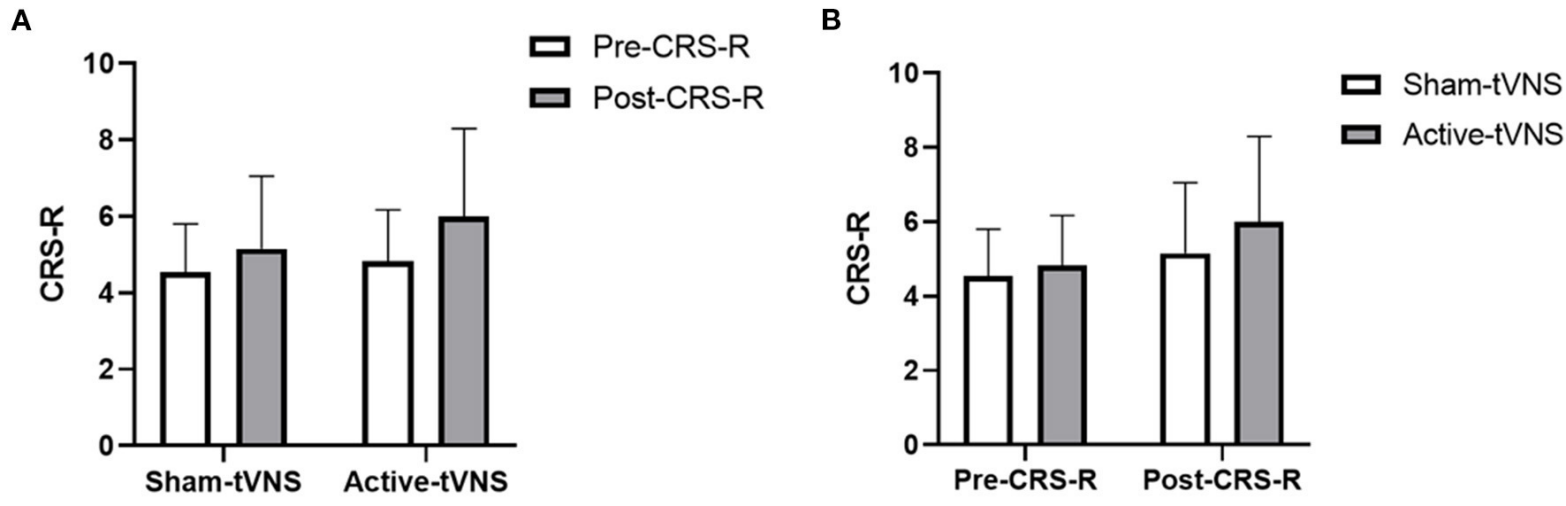
**(A)** Coma Recovery Scale-Revised (CRS-R) improvement in VS/UWS subject before and after treatment in the groups. **(B)** Comparison of CRS-R improvement VS/UWS subject between the two groups before and after treatment.

The total CRS-R scores improved in both the active- and sham-tVNS groups compared with the baseline scores. Active tVNS stimulation produced greater improvement in the total CRS-R score than that with the sham treatment, although not statistically significant (10.93 ± 4.99 vs. 9.28 ± 4.38, *P* = 0.198) (Figure 2). Although the results demonstrated that active tVNS induced improved consciousness in patients with DoCs compared with that at baseline (10.93 ± 4.99 vs. 8.96 ± 4.02, *P* = 0.111), we found that not all patients improved. Specifically, 85.71% (24/28) of patients benefited from tVNS treatment, and the CRS-R scores increased by 0 points, 1 point, 2 points, and 3 points or more in four, five, nine, and nine patients, respectively, in the active-tVNS group (Figure 3). Further analysis of the active-tVNS group revealed that all patients with MCS improved; however, only 66.67% (8/12) patients with VS/UWS improved. Consequently, we then evaluated the responses of patients with MCS in the two groups after active (16 patients) or sham (16 patients) tVNS. We found that the CRS-R scores of patients with MCS improved significantly after active tVNS stimulation compared with sham stimulation (12.06 ± 1.98 vs. 14.63 ± 2.63, *P* = 0.004) (Figure 4). The CRS-R scores of patients with MCS in the active-tVNS group were significantly higher after stimulation compared with those in the sham-tVNS group after sham stimulation (14.63 ± 2.63 vs. 12.62 ± 2.50, *P* = 0.035) (Figure 4). No obvious difference was observed in patients with VS/UWS after active or sham stimulation (6.00 ± 2.30 vs. 5.15 ± 1.91, *P* = 0.330) (Figure 5).

## 4. Discussion

This randomized, double-blinded, sham-controlled study investigated whether tVNS could arouse consciousness in patients with DoCs. Our data demonstrate that tVNS improves the consciousness of patients with DoCs. However, not all patients with DoCs benefited from tVNS treatment, although the CRS-R scores of all patients with MCS c improved after tVNS stimulation. In addition, no obvious side effects related to tVNS were reported or observed.

Restoring the lives of patients with chronic DoCs has long been a focus and challenge in medical research. Due to the limited effectiveness of existing drugs, recent research has increasingly focused on neuromodulatory therapies such as median nerve stimulation (14, 15), transcranial direct current stimulation (16), transcranial magnetic stimulation (17), deep brain stimulation (18) and so on. Additional treatment methods should be explored owing to the lack of strong medical evidence and because the above techniques have inconsistent therapeutic effects.

Vagus nerve stimulation is a neuromodulatory technique that has been widely used to manage a number of clinical disorders, such as epilepsy (19), heart failure (20), depression (21) and inflammatory bowel disease (22), its therapeutic potential in human patients with DoCs has been discussed. Recently, several studies, including two case reports and one case series, provided early evidence supporting that vagus nerve stimulation improves consciousness levels in patients with DoCs. Corazzol et al. (8) performed surgical vagus nerve stimulation in a patient with VS/UWS who remained in this status for more than 15 years. Improvements in arousal, attention, and visual function were observed in the patient during the 6-month treatment regimen, accompanied by enhanced cortical functional connectivity and increased thalamic metabolic activity. Researchers in China (7) performed tVNS on an older woman with VS/UWS for 50 days after cardiopulmonary resuscitation. Improved behavioral responses and increased functional connections among several brain regions indicated an improved state of consciousness after tVNS. In addition, a case series reported (9) that after 4 weeks of tVNS treatment and another 4-week follow-up, behavioral improvements were observed in five of eight patients with MCS whose conscious states had been maintained for over 4 weeks before stimulation, thereby indicating the long-lasting therapeutic potential of tVNS in patients with MCS. However, randomized controlled trials are still required to test the efficiency of arousal by tVNS in patients with DoCs. Accordingly, we used sham-controlled designs in the present study to demonstrate the therapeutic efficacy of tVNS in promoting the recovery of consciousness in patients with DoCs.

tVNS is a non-invasive method to modulate nerve activity by delivering an electrical current to the auricular nail *via* the vagal reflex (23). The possible mechanisms that contribute to tVNS promoting these effects in patients with DoCs have been explored. First, tVNS produces widespread brain activity changes in brain regions such as the solitary nucleus, locus coeruleus, raphe nuclei, insula, and sensory cortex in patients with DoCs (24, 25). Second, investigators found that vagal afferents activated the ascending reticular activating system, which is vital for promoting and sustaining a conscious state, from the periaqueductal gray and raphe nuclei to the thalamus (26–28). In addition, tVNS increased brain activity and connectivity within the external network through norepinephrine and orexin/hypocretin pathways (10, 29). We believe that these mechanisms provide evidence for the therapeutic efficacy of tVNS in neurological injury, and that the present study provides preliminary evidence that tVNS improves consciousness in some patients with DoCs.

Consciousness is described as a combination of two components: arousal and awareness (30, 31). Arousal refers to alertness or degree of alertness whereas awareness refers to the ability to interact with the environment or self. VS/UWS is defined as a lack of awareness of the environment or self-observed at the bedside, despite the presence of intermittent wakefulness (awakening), either spontaneous or following tactile, auditory, or painful stimuli (32, 33). Patients with MCS are characterized by changes in arousal levels and the recovery of fluctuations, although with repeatable signs of awareness such as visual pursuit, target orientation, or command following (34, 35). In the present study, we found that tVNS had different arousal effects in patients with VS/UWS and MCS. Patients with MCS in the active-tVNS group were significantly improved after tVNS stimulation compared with patients in the sham-tVNS group after sham stimulation. The CRS-R scores of all patients with MCS improved (1 point, 2 points, and 3 points or more in 1 patient, 7 patients, and 8 patients, respectively) in the active-tVNS group; however, only 58.33% (7/12) of patients with VS/UWS improved in the active-tVNS group. In addition, patients with VS/UWS showed no significant difference compared with the sham-tVNS group after active or sham tVNS stimulation. The reasons patients with MCS and patients with VS/UWS responded differently to tVNS are unclear, further studies with large samples of patients are therefore required.

This study had several limitations. Further multicenter studies with larger sample sizes are needed to support our conclusions. We will recruit a greater number of patients with DoCs with different etiologies from multiple hospitals to test the effects of tVNS in future studies. Furthermore, the methods of DoC diagnosis should be considered, such as electroencephalogram, evoked potentials, and functional magnetic resonance imaging. In addition, patients with MCS and VS/UWS may respond to different tVNS protocols that were not used in the present study. In the future, we will include different tVNS parameters in different types of patients with DoCs to find a better scheme.

In conclusion, our study provides preliminary evidence of the therapeutic efficacy of tVNS for promoting improved consciousness in patients with DoCs, especially patients with MCS. However, not everyone benefits from this procedure. Therefore, a prediction model that combines etiology, with the portion of the brain that is injured as well as other factors should be developed in the future to identify patients with DoCs prior to the clinical application of tVNS. Furthermore, identifying the optimal tVNS protocol is key for achieving significant effects. As no convincing evidence of the optimal tVNS protocol for treating patients with DoCs exists, additional research is required.

## Data availability statement

The original contributions presented in the study are included in the article/Supplementary material, further inquiries can be directed to the corresponding authors.

## Ethics statement

The studies involving human participants were reviewed and approved by the Ethics Committee of the First Affiliated Hospital of Nanchang University. The patients/participants provided their written informed consent to participate in this study.

## Author contributions

Y-FZ: data collection and curation, formal analysis, and writing—original draft. J-WK: data curation, resources, and writing—original draft. QX: data curation and data analysis. ZF: conceptualization, supervision, and review the draft. X-YD: data curation and analysis, supervision, writing—review, and editing the original draft. All authors contributed to the article and approved the final version.
