# Supplementary material for: Transauricular vagus nerve stimulation for patients with disorders of consciousness: A randomized controlled clinical trial
Source: Front Neurol. 2023 Mar 2;14:1133893. doi: 10.3389/fneur.2023.1133893 (PMC10017768; doi:10.3389/fneur.2023.1133893)
Supplement: Supplementary file 1 [file Table_1.docx]

**Supplementary Table 1** Detailed information for all patients.

| **ID** | **Group allocation** | **Age (yr)** | **Sex** | **Duration of disease (days)** | **Etiology** | **Coma Recovery Scale-Revised score** | |
| --- | --- | --- | --- | --- | --- | --- | --- |
|  |  |  |  |  |  | **Pre** | **Post** |
| 1 | Sham-tVNS | 55 | Male | 143 | TBI | 7 (VS/UWS) | 9 (MCS) |
| 2 | Active-tVNS | 69 | Male | 128 | Stroke | 15 (MCS) | 18 (EMCS) |
| 3 | Sham-tVNS | 45 | Male | 126 | TBI | 6 (VS/UWS) | 7 (VS/UWS) |
| 4 | Active-tVNS | 49 | Male | 136 | TBI | 14 (MCS) | 18 (EMCS) |
| 5 | Sham-tVNS | 74 | Female | 115 | TBI | 13 (MCS) | 14 (MCS) |
| 6 | Active-tVNS | 50 | Male | 149 | Stroke | 14 (MCS) | 17 (EMCS) |
| 7 | Sham-tVNS | 55 | Male | 128 | TBI | 4 (VS/UWS) | 5 (VS/UWS) |
| 8 | Active-tVNS | 57 | Male | 112 | Stroke | 13 (MCS) | 16 (EMCS) |
| 9 | Sham-tVNS | 51 | Female | 110 | Stroke | 5 (VS/UWS) | 5 (VS/UWS) |
| 10 | Active-tVNS | 65 | Female | 105 | Stroke | 13 (MCS) | 15 (MCS) |
| 11 | Sham-tVNS | 64 | Female | 136 | TBI | 13 (MCS) | 13 (MCS) |
| 12 | Active-tVNS | 55 | Female | 109 | Stroke | 12 (MCS) | 15 (MCS) |
| 13 | Sham-tVNS | 53 | Female | 103 | Stroke | 3 (VS/UWS) | 3 (VS/UWS) |
| 14 | Active-tVNS | 68 | Female | 109 | Stroke | 11 (MCS) | 14 (MCS) |
| 15 | Sham-tVNS | 43 | Male | 111 | Stroke | 4 (VS/UWS) | 4 (VS/UWS) |
| 16 | Active-tVNS | 43 | Male | 117 | TBI | 11 (MCS) | 13 (MCS) |
| 17 | Sham-tVNS | 55 | Male | 117 | TBI | 3 (VS/UWS) | 3 (VS/UWS) |
| 18 | Active-tVNS | 59 | Female | 139 | Stroke | 10 (MCS) | 13 (MCS) |
| 19 | Sham-tVNS | 55 | Female | 138 | Stroke | 11 (MCS) | 12 (MCS) |
| 20 | Active-tVNS | 55 | Male | 112 | Stroke | 10 (MCS) | 12 (MCS) |
| 21 | Sham-tVNS | 43 | Male | 120 | Stroke | 15 (MCS) | 15 (MCS) |
| 22 | Active-tVNS | 59 | Female | 114 | Stroke | 7 (VS/UWS) | 10 (MCS) |
| 23 | Sham-tVNS | 55 | Male | 125 | TBI | 9 (MCS) | 9 (MCS) |
| 24 | Active-tVNS | 44 | Male | 144 | TBI | 6 (VS/UWS) | 9 (MCS) |
| 25 | Sham-tVNS | 61 | Female | 140 | Stroke | 12 (MCS) | 12 (MCS) |
| 26 | Active-tVNS | 50 | Male | 103 | Stroke | 5 (VS/UWS) | 6 (VS/UWS) |
| 27 | Sham-tVNS | 55 | Female | 118 | Stroke | 10 (MCS) | 11 (MCS) |
| 28 | Active-tVNS | 43 | Male | 100 | TBI | 5 (VS/UWS) | 5 (VS/UWS) |
| 29 | Sham-tVNS | 52 | Male | 90 | Stroke | 11 (MCS) | 11 (MCS) |
| 30 | Active-tVNS | 73 | Female | 126 | TBI | 4 (VS/UWS) | 4 (VS/UWS) |
| 31 | Sham-tVNS | 59 | Female | 136 | TBI | 15 (MCS) | 16 (MCS) |
| 32 | Active-tVNS | 56 | Female | 124 | Stroke | 3 (VS/UWS) | 3 (VS/UWS) |
| 33 | Sham-tVNS | 74 | Female | 169 | TBI | 16 (MCS) | 18 (EMCS) |
| 34 | Active-tVNS | 67 | Female | 102 | TBI | 7 (VS/UWS) | 9 (MCS) |
| 35 | Sham-tVNS | 59 | Female | 116 | Stroke | 14 (MCS) | 15 (MCS) |
| 36 | Active-tVNS | 75 | Male | 104 | Stroke | 5 (VS/UWS) | 6 (VS/UWS) |
| 37 | Sham-tVNS | 55 | Female | 130 | TBI | 13 (MCS) | 13 (MCS) |
| 38 | Active-tVNS | 53 | Female | 109 | TBI | 12 (MCS) | 14 (MCS) |
| 39 | Sham-tVNS | 55 | Female | 116 | Stroke | 12 (MCS) | 12 (MCS) |
| 40 | Active-tVNS | 57 | Female | 118 | Stroke | 9 (MCS) | 10 (MCS) |
| 41 | Sham-tVNS | 73 | Male | 100 | Stroke | 11 (MCS) | 12 (MCS) |
| 42 | Active-tVNS | 59 | Male | 113 | TBI | 12 (MCS) | 14 (MCS) |
| 43 | Sham-tVNS | 49 | Male | 104 | TBI | 10 (MCS) | 10 (MCS) |
| 44 | Active-tVNS | 53 | Male | 130 | Stroke | 16 (MCS) | 20 (EMCS) |
| 45 | Sham-tVNS | 67 | Male | 150 | TBI | 9 (MCS) | 9 (MCS) |
| 46 | Active-tVNS | 55 | Female | 121 | Stroke | 3 (VS/UWS) | 4 (VS/UWS) |
| 47 | Sham-tVNS | 54 | Female | 130 | Stroke | 7 (VS/UWS) | 8 (VS/UWS) |
| 48 | Active-tVNS | 45 | Male | 103 | Stroke | 5 (VS/UWS) | 7 (VS/UWS) |
| 49 | Sham-tVNS | 55 | Male | 122 | TBI | 5 (VS/UWS) | 6 (VS/UWS) |
| 50 | Active-tVNS | 59 | Male | 97 | TBI | 4 (VS/UWS) | 5 (VS/UWS) |
| 51 | Sham-tVNS | 69 | Female | 170 | TBI | 5 (VS/UWS) | 5 (VS/UWS) |
| 52 | Active-tVNS | 56 | Female | 145 | Stroke | 4 (VS/UWS) | 4 (VS/UWS) |
| 53 | Sham-tVNS | 40 | Male | 133 | TBI | 4 (VS/UWS) | 5 (VS/UWS) |
| 54 | Active-tVNS | 31 | Male | 140 | TBI | 10 (MCS) | 12 (MCS) |
| 55 | Sham-tVNS | 74 | Male | 107 | TBI | 4 (VS/UWS) | 4 (VS/UWS) |
| 56 | Active-tVNS | 62 | Male | 92 | TBI | 11 (MCS) | 13 (MCS) |
| 57 | Sham-tVNS | 62 | Female | 102 | Stroke | 3 (VS/UWS) | 3 (VS/UWS) |

Abbreviations: tVNS, transcutaneous vagus nerve stimulation; EMCS, emergence of the minimally conscious state; MCS, minimally conscious state; VS/UWS, vegetative state/unresponsive wakefulness Syndrome;
